# Human amnionic progenitor cell secretome mitigates the consequence of traumatic optic neuropathy in a mouse model

**DOI:** 10.1016/j.omtm.2023.04.002

**Published:** 2023-04-18

**Authors:** Robyn McCartan, Arissa Gratkowski, Mackenzie Browning, Coral Hahn-Townsend, Scott Ferguson, Alexander Morin, Corbin Bachmeier, Andrew Pearson, Larry Brown, Michael Mullan, Fiona Crawford, Radouil Tzekov, Benoit Mouzon

**Affiliations:** 1 Roskamp Institute, 2040 Whitfield Avenue, Sarasota, FL 34243, USA; 2 Bay Pines Veterans’ Hospital, Saint Petersburg, FL 33708, USA; 3 James A. Haley Veterans’ Hospital, Tampa, FL 33612, USA; 4 Noveome Biotherapeutics, Inc., Pittsburgh, PA 15219, USA; 5 University of South Florida, Tampa, FL 33620, USA; 6 University of Miami Miller School of Medicine, Miami, FL 33136, USA

**Keywords:** traumatic optic neuropathy, optic nerve trauma,, retinal ganglion cells, traumatic brain injury, neurotrauma, sectretome, mouse model

## Abstract

Traumatic optic neuropathy (TON) is a condition in which acute injury to the optic nerve from direct or indirect trauma results in vision loss. The most common cause of TON is indirect injury to the optic nerve caused by concussive forces that are transmitted to the optic nerve. TON occurs in up to 5% of closed-head trauma patients and there is currently no known effective treatment. One potential treatment option for TON is ST266, a cell-free biological solution containing the secretome of amnion-derived multipotent progenitor (AMP) cells. We investigated the efficacy of intranasal ST266 in a mouse model of TON induced by blunt head trauma. Injured mice treated with a 10-day regimen of ST266 showed an improvement in spatial memory and learning, a significant preservation of retinal ganglion cells, and a decrease in neuropathological markers in the optic nerve, optic tract, and dorsal lateral geniculate nucleus. ST266 treatment effectively downregulated the NLRP3 inflammasome-mediated neuroinflammation pathway after blunt trauma. Overall, treatment with ST266 was shown to improve functional and pathological outcomes in a mouse model of TON, warranting future exploration of ST266 as a cell-free therapeutic candidate for testing in all optic neuropathies.

## Graphical abstract

**Figure:**
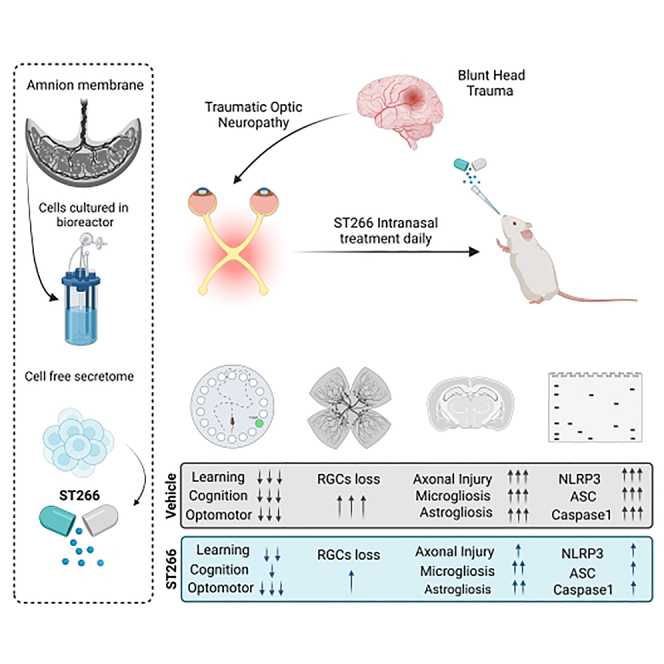

## Teaser

McCartan and colleagues found that a potential treatment option for traumatic optic neuropathy (TON) is the secretome of amnion-derived multipotent progenitor cells (ST266). In this study using a mouse model of TON, it was found that ST266 treatment mitigated neuroinflammation and retinal ganglion cells loss, demonstrating its potential as a cell-free therapeutic candidate for all optic neuropathies.

## Introduction

Traumatic optic neuropathy, or TON, is an injury to the optic nerve as a result of indirect or direct trauma to the head.^1^ Direct injury occurs when the head experiences trauma that results in lacerations to the optic nerve causing severe vision loss and a low chance of recovery.^1^^,^^2^ Indirect injury, and the focus of this study, is caused by blunt force trauma resulting in neuroinflammation that subsequently causes damage to the optic nerve accompanied by mild to severe vision loss.^1^^,^^2^^,^^3^^,^^4^ There is an observable association between TON and traumatic brain injuries as a result of falls, car accidents, and assaults, which occurs in 0.5%–5% of closed-head trauma patients.^1^^,^^2^ The pathogenesis of indirect TON has not been fully elucidated, and due to a lack of clear guidelines, there is much ambiguity regarding its diagnosis and treatment.

Current management for TON includes clinical observation or optic canal decompression (with or without corticosteroids) to minimize damage.^5^^,^^6^^,^^7^^,^^8^^,^^9^ Evidence that corticosteroids are effective in the treatment of central nervous system injury has come from different animal models of spinal cord^10^^,^^11^ and traumatic brain injury.^12^ However, clinical evidence that corticosteroids improve TON and the survival of retinal ganglion cells (RGCs) is lacking. Furthermore, there are also several animal studies demonstrating that corticosteroids have pro-apoptotic effects on RGCs^13^^,^^14^ and exacerbate neuroinflammation and axonal injury in the optic nerve.^15^

**Figure 1:**
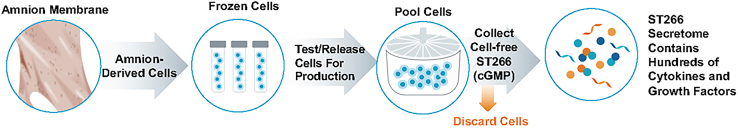
ST266 production description

Cell-free therapies have become a topic of interest in the development of new therapeutics.^16^ Both preclinical and clinical studies have shown the anti-inflammatory and anti-apoptotic therapeutic potential of ST266 (formerly ACCS, amnion-derived cellular cytokine solution),^17^^,^^18^^,^^19^^,^^20^^,^^21^^,^^22^^,^^23^^,^^24^ a biologic solution containing secreted products from proprietary amnion-derived multipotent progenitor (AMP) cells (Figure 1). The experimental autoimmune encephalomyelitis (EAE) model of multiple sclerosis results in progressive loss of RGCs, but in a mouse study, intranasal delivery of ST266 showed recovery of visual acuity, preservation of RGCs, and reduced demyelination of the optic nerve.^18^^,^^19^

**Figure 2:**
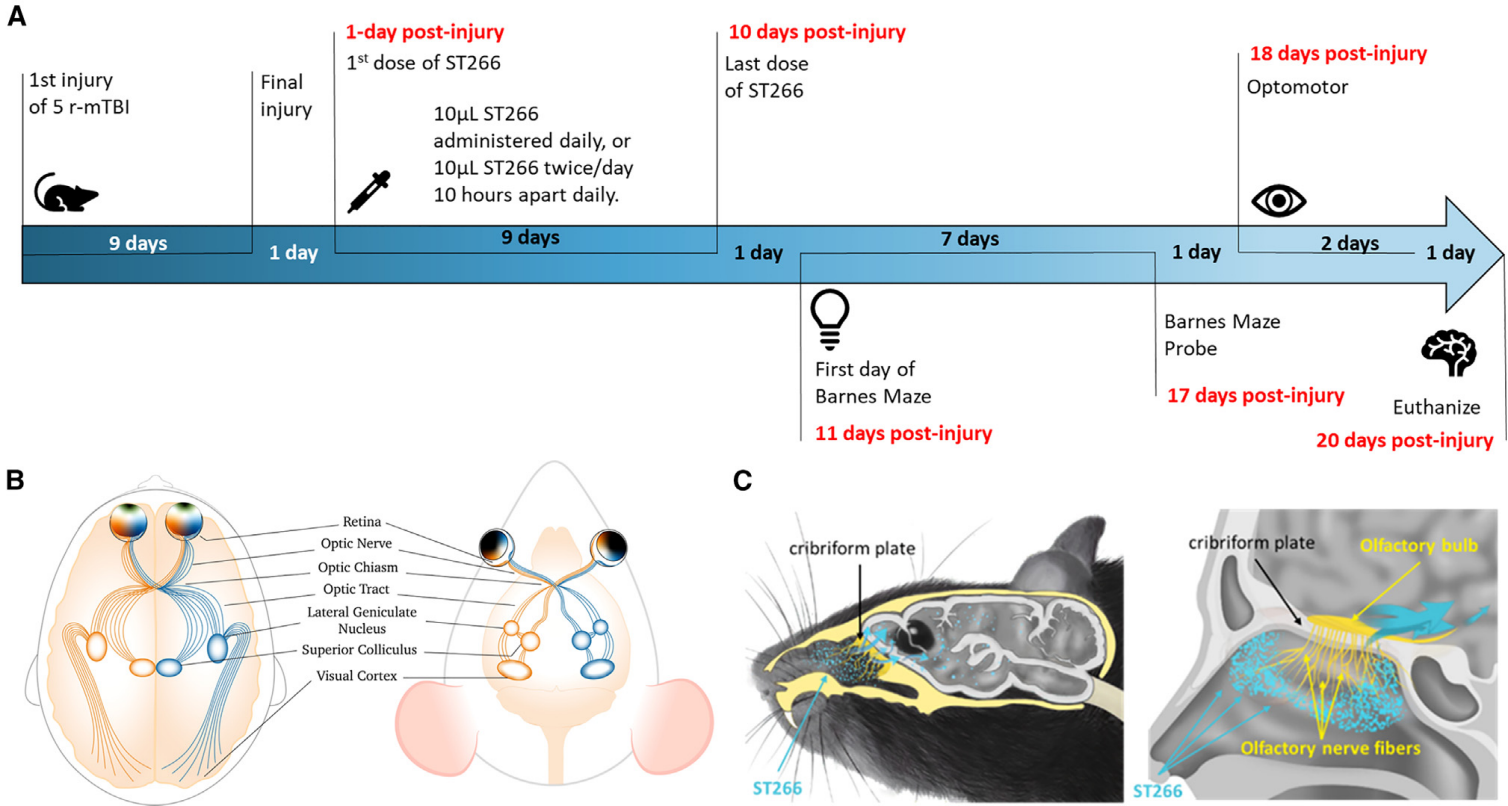
Schematic of the experimental design and comparison of anatomical visual features between humans and mice (A) Outline of experimental schedule. (B) A comparison of the anterior and posterior visual pathways between humans and mice is demonstrated, since dysfunction in both pathways is a key aspect of TON pathology. (C) Nose to brain route of administration of ST266 to the central nervous system in a mouse and in human bypassing the blood-brain barrier in the process.

We investigated the efficacy of ST266 as a cell-free therapeutic to treat TON in our mouse model of closed-head injury^25^^,^^26^^,^^27^ (Figure 2), which has demonstrated progressive neuropathological changes to the visual system at both acute and chronic time points.^28^^,^^29^^,^^30^^,^^31^ Healing following TON is limited, as the injury site in the optic nerve is composed of dead cell bodies and debris that are unable to be phagocytosed, ultimately impairing the communication lines in the nerve. If healing does occur, it is likely limited to the periphery of the injury site and involves stimulating a cascade of multiple growth factors and cytokines. The ST266 secretome contains hundreds of growth factors and cytokines that simultaneously or sequentially up- and downregulate numerous critical cellular pathways.^20^ Selecting the optic nerve as a target for treatment is strongly supported by the observation of radiolabeled ST266 in the optic nerve after intranasal injection and subsequent demonstration of treatment efficacy in a mouse model of EAE.^18^ Overall, the results from our study support ST266 as a potential therapy for TON, as it reduces RGC loss, improves learning and spatial memory, and alleviates the ongoing neuroinflammation in the visual pathway.

## Results

### ST266 improved learning and cognitive performance but did not affect optomotor response deficit after TON

**Figure 3:**
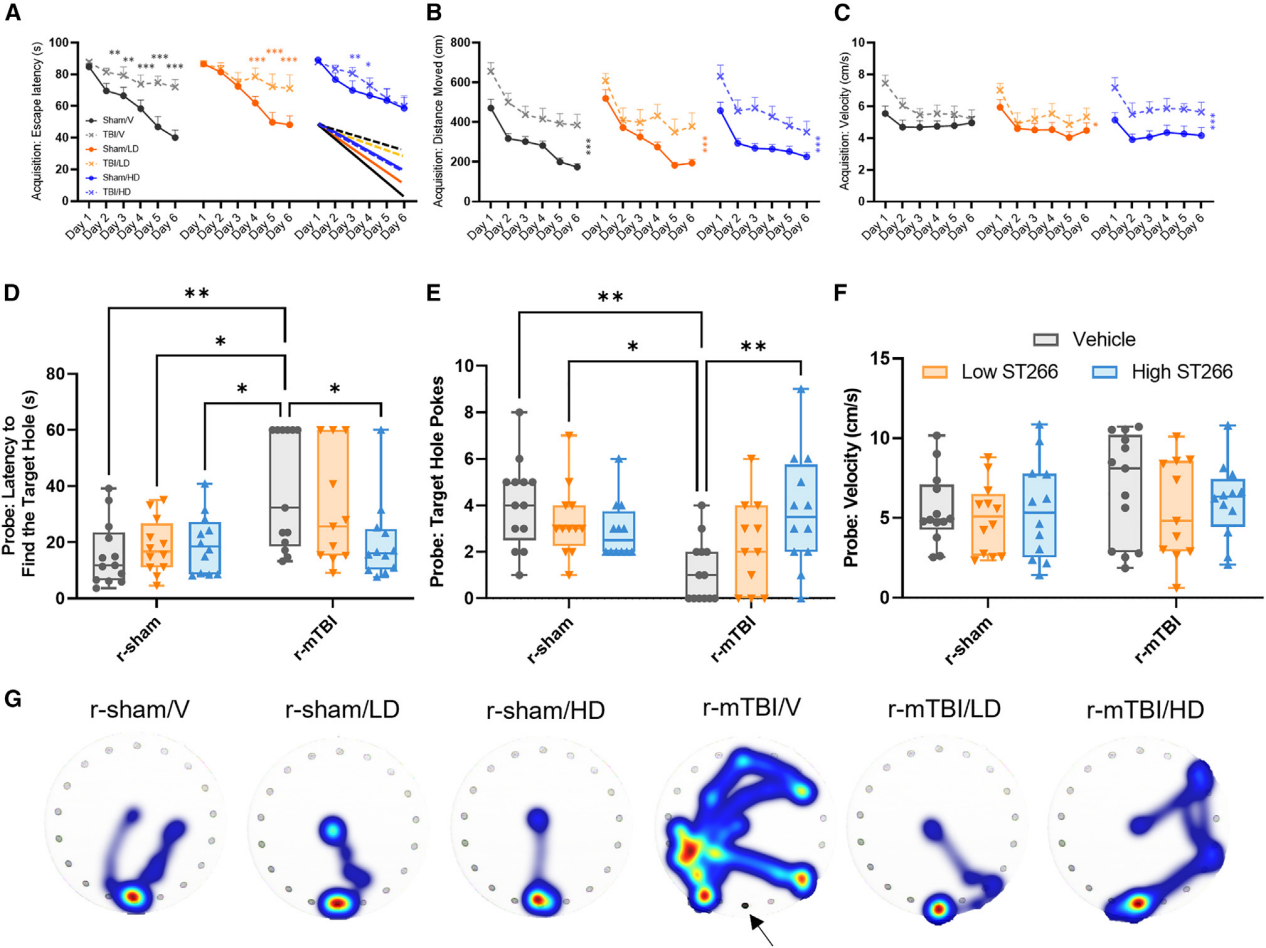
Evaluation of learning (acquisition) and spatial memory retention (probe) using the Barnes maze on days 11–17 after first mild traumatic brain injury (mTBI) Mice were tested in the Barnes maze for their ability to locate a black box at the target hole and escape the maze. (A) During the acquisition testing, the r-mTBI/V and the r-mTBI/LD injured spent more time on the table before escaping into the target hole compared with their respective controls. The r-mTBI/HD group performance was similar to that of their respective control, the r-sham/HD. The groups differed on the last day of acquisition; both r-mTBI/V and r-mTBI/LD failed to escape to the target hole compared with their sham controls on days 4, 5, and 6 of acquisition; Wilcoxon signed-rank test, ∗p < 0.05; ∗∗p < 0.001; ∗∗∗p < 0.0001. No difference was observed between the r-mTBI/HD and r-sham/HD, but the r-sham/HD performed significantly worse for the 6 days of acquisition; Wilcoxon signed-rank test, ∗∗∗p < 0.0001. (B) The distance traveled on the table was the longer than that of the sham groups and similar for all injured groups. There is an injury effect between r-sham/V and r-mTBI/V, r-sham/LD and r-mTBI/LD, and r-sham/HD and r-mTBI/HD; ∗∗∗p < 0.0001; repeated-measures ANOVA. No treatment effect was observed across the r-mTBI groups or across their respective controls. (C) The mean velocity for acquisition phase of the two r-mTBI-treated groups was always higher than that of their respective sham controls. The mean velocity was similar across all sham groups. The mean velocity of the r-mTBI/HD was always higher than that of all groups, ∗p < 0.05; ∗∗∗p < 0.0001 repeated-measures ANOVA. (D) For the probe trial (1 day following the 6 days of acquisition testing), the target box was removed, and mice were placed in the middle of the table for a single 60 s trial. The r-mTBI/V had a greater latency to reach the target hole than their respective control (r-sham/V vs. r-mTBI/V; ∗p < 0.05; ∗∗p < 0.001; two-way ANOVA with Tukey’s multiple comparisons test). There was no difference between the r-sham/HD and the r-mTBI/HD group or between the r-sham/LD and the r-mTBI/LD (p > 0.05; two-way ANOVA with Tukey’s multiple comparisons test). (E) Of all groups, only the r-mTBI/V showed a reduction in target hole nose poke (r-sham/V vs. r-mTBI/V, ∗∗p < 0.001; two-way ANOVA with Tukey’s multiple comparisons test. (F) The mean velocity and distance traveled for probe test was similar across all groups. (G) Heatmap images showing the cumulative time spent within the maze during probe day (day 7; arrow points to the location of the target box). Data are presented as mean ± SEM; each symbol represents one mouse. For clarity on the graphs, only significant results between the groups are reported.

Barnes maze testing was performed on days 11 through 17 after first mild traumatic brain injury (mTBI). Acquisition trial testing showed an effect of injury on spatial learning (Figure 3A) that was diminished proportional to the dose of treatment, with the high-dose animal performing similar to the respective control, except on acquisition days 3 and 4. An injury effect was observed for the distance moved during the acquisition trials, but there was no treatment effect (Figure 3B). Walking speed was increased in all injured animals, but velocity was not affected by treatment (Figure 3C). On day 7, probe test performance was markedly impaired in the repetitive mTBI (r-mTBI)-vehicle group compared with the r-mTBI-high-dose group, suggesting a beneficial effect of ST266 by improving spatial memory learning (Figure 3D). The number of exploratory nose pokes into the target hole was also restored to sham control level after a high-dose treatment with ST266 (Figure 3E). The mean velocity for the probe trial was similar across all groups (Figure 3F). Analyses of the Barnes maze tracks on probe day (day 7) revealed that the travel path of the r-mTBI animals was more circuitous, searching in all quadrants compared with the path of the shams or the treated injured animals (Figure 3G). Finally, an optomotor test was performed on day 18 after the first mTBI to evaluate visual defects. Optomotor response was assessed using repeating stripes at a speed of 2 rpm. Stripe thickness varied from 0.25 to 0.9 cycles per degree (CPD). The optomotor response frequency and duration significantly decreased with increasing CPD and in response to injury, but no treatment effect was reported (Figures S1 and S2).

### ST266 reduced the loss of retinal ganglion cells at a subacute time point post TON

**Figure 4:**
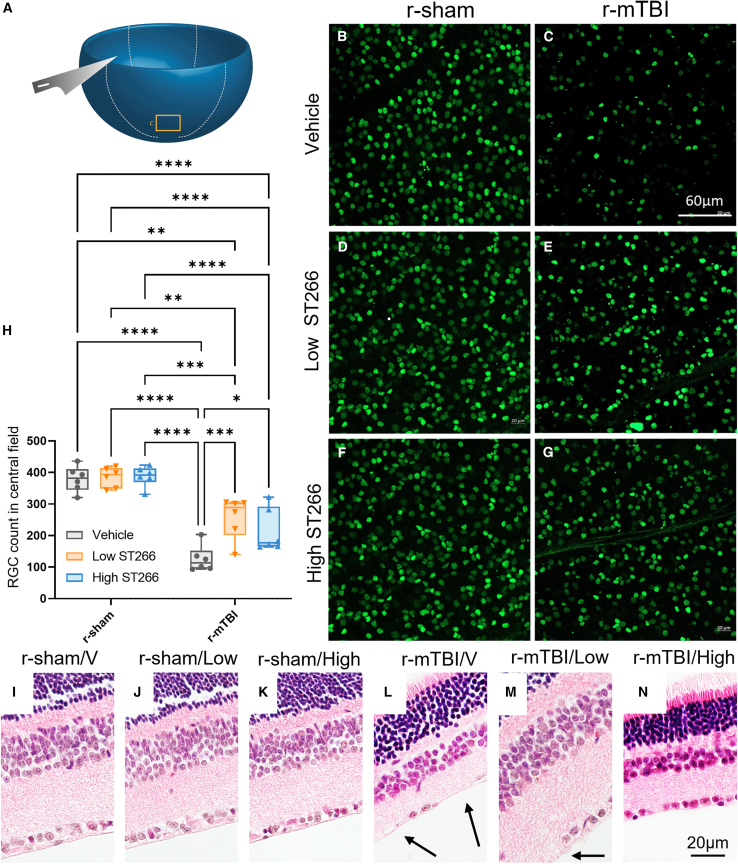
ST266 reduced the loss of retinal ganglion cells at 20 days post first injury/anesthesia Representative images of retina flat mounts. (A) Diagram shows the location where the standardized photos of RGCs were obtained in the central region (rectangular box) of each retinal quadrant. (B–G) Representative images of Brn3a-immunolabeled RGCs from the central region for each treatment group. (B–G) Visual inspection and (H) quantitative data from RGC counting demonstrated a reduced cellular density on retinas after r-mTBI compared with retinas after r-sham. Eyes from brain-injured animals treated with ST266 showed greater RGC counts than vehicle-treated animals. Data are presented as box and whisker plots: min to max (all points are shown; symbol represents one mouse, n = 6 per group). Two-way ANOVA summary revealed an overall injury and treatment effect for the dataset: injury F(1, 30) = 126.9, p < 0.0001; treatment F(2, 30) = 6.297, p = 0.052; treatment F(1, 75) = 31.50, p < 0.0001. Tukey’s multiple comparisons are represented on the graph ∗p < 0.05; ∗∗p < 0.001; ∗∗∗p < 0.0001; ∗∗∗∗p < 0.00001. (I–N) Representative cross sections of equivalent areas in the upper retina from (I–K) sham mice and (L–N) mice after r-mTBI stained with H&E. Areas of reduced cellular density in the RGC layer are indicated by arrows. (J and N) Low and high dose of ST266 prevented cell loss in the retinal layers.

RGCs, the main projection neurons in the optic nerve/optic tract, were examined for evidence of neuronal degeneration in sham animals and animals subjected to injury. RGC loss in the central field after TON in whole-mount retinas was obvious at 19 days after the first mTBI (Figure 4H). Mice injured and treated daily for a period of 10 days with either high- or low-dose ST266 had higher RGC densities than the vehicle-treated group. (Figure 4H). There was no difference in RGC densities between the sham groups. Cross sections and analysis of the cellularity in the RGC layer with H&E staining corroborate the results observed via Brn3a immunolabeling, demonstrating a reduction in RGC count in sham groups alleviated by ST266 administration in the injured groups (Figures 4I–N).

### ST266 reduced reactive gliosis and axonal damage in the area of secondary damage but not in the primary injury locus

**Figure 5:**
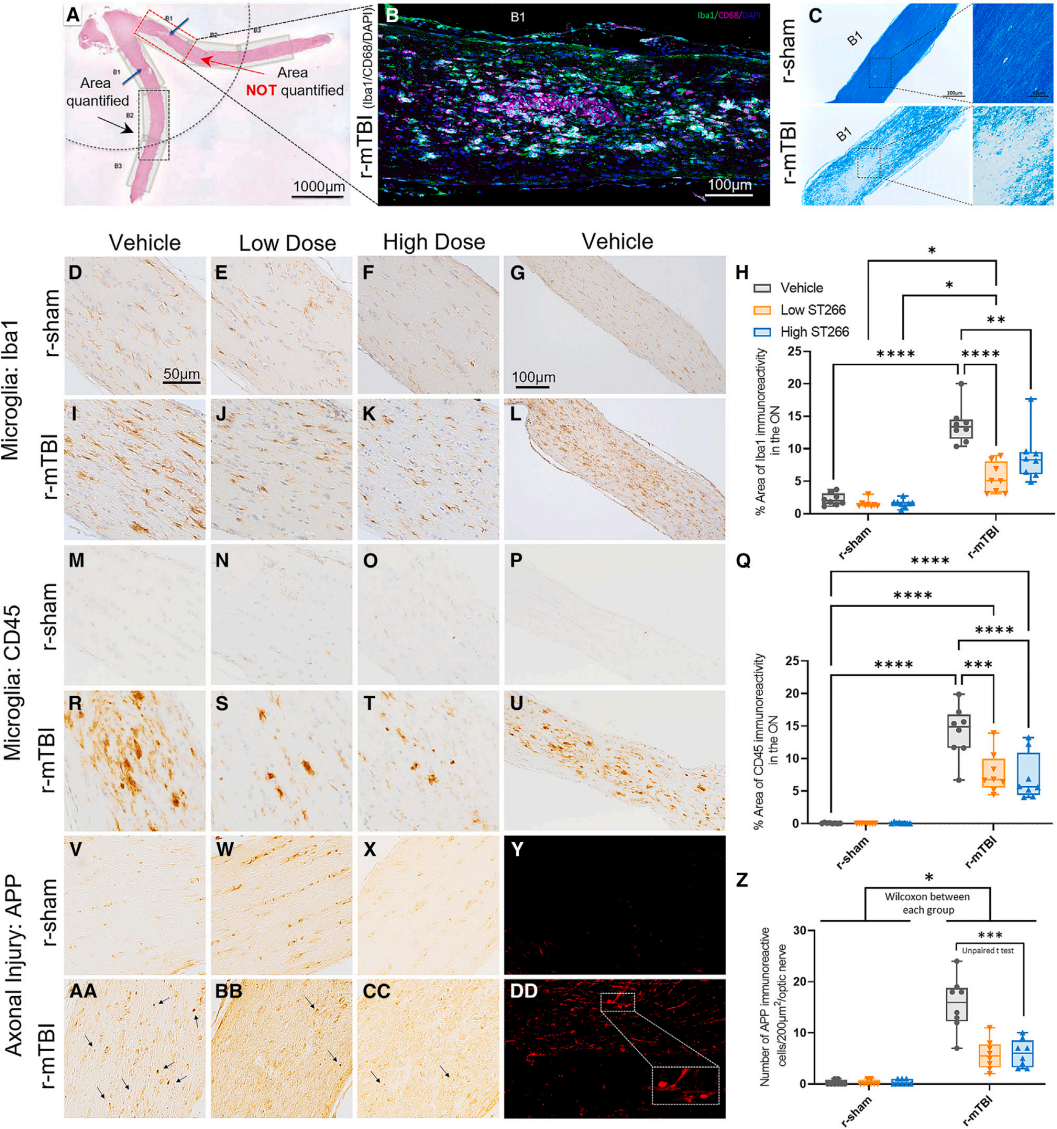
Treatment with ST266 attenuated microgliosis and axonal injury in the optic nerve (A) Longitudinal cross section of optic nerves at 20 days post first injury (H&E stain). Only area B2 was part of the quantification analysis shown in (H), (Q), and (AA). Area B1, closest to the chiasm in (A), was excluded from the analysis as no treatment effect was observed in this region, where most of the focal cellular damage was located (B and C). (B) Dual immunofluorescence assessment of Iba1 expression (green) and CD68 (purple) in the B1 region of the optic nerve; DAPI (blue) was used to label the nucleus. The presence of foamy/phagocytic macrophages was observed in this area of high cellularity and demyelination, as shown in (C) with a Luxol fast blue stain. A sagittal section of the optic nerve was immunostained with (D–L) Iba1 and (M–U) cluster differentiation 45 (CD45) to assess microgliosis. (H and Q) Both low and high doses of ST266 reduced Iba1 and CD45 immunoreactivity in the optic nerve. For Iba1, a two-way ANOVA revealed an overall injury and treatment effect: injury F(1, 42) = 131.8, p < 0.0001; treatment F(2, 42) = 15.56, p < 0.0001. For CD45, an overall injury and treatment effect was also present: injury F(1, 42) = 172.1, p < 0.0001; treatment F(2, 42) = 9.11, p = 0.0005. Tukey’s multiple comparisons are represented on the graphs ∗∗∗p < 0.0001; ∗∗∗∗p < 0.00001. (V–EE) A sagittal section of the optic nerve was immunostained with a marker of axonal damage (APP). Immunoreactive axonal profiles were observed as granular (arrows) and occasionally more elongated fusiform, as represented in (DD) via immunofluorescence with APP (red). (V and X–Y) No APP staining was observed in the sham animals. (Z) ST266 treatment reduced the number granular positive axons after r-mTBI ∗p < 0.05; ∗∗∗p < 0.0001. Tissue sections were counterstained with hematoxylin. Data are presented as box and whisker plots: min to max (all points are shown; symbol represents one mouse, n = 8 per group). For APP analysis the data were still not normal after transformation; nonparametric methods were used for analysis; unpaired t test, n = 8, ∗∗∗p < 0.001 r-mTBI/V vs. r-mTBI-low or high ST266.

To assess the changes in demyelination, inflammation, and neurodegeneration in the optic nerve, longitudinal optic nerve sections from each group were evaluated using routine histological methods, immunohistochemistry (IHC) and immunofluorescence (IF). As expected from our previous mTBI studies on the visual system,^29^^,^^30^^,^^31^ topographical differences in degree of damage/cellularity were noted along the optic nerve. The cellularity was much more pronounced in the area containing “cavernous” degeneration, within 1,000 μm of the chiasm, compared with areas farther away from the chiasm (Figure 5A). For a more precise analysis, we chose to split each optic nerve into three equal areas in length as follows: B1, B2, and B3. Area B1 was the closest to the optic chiasm and contained the area with the primary damage, highest cellularity, and “cavernous” degeneration. Area B2 was anterior to B1, ending at the approximate location of the optic nerve entry into the orbit canal, and B3 represented most of the length of the intraorbital part of the nerve.

As with our previous studies,^29^^,^^30^^,^^31^ B1 was the zone where the most macroscopic and cellular damage was observed following injury (Figures 5B and 5C). Increased cellularity, cavernous degeneration, areas of demyelination, and reactive gliosis were observed in all groups regardless of the treatment dose. Morphologic evidence of cavernous degeneration and increased cellularity with the presence of activated microglia was revealed by triple confocal staining with ionized calcium binding adaptor molecule 1 (Iba1), cluster of differentiation 68 (CD68), and DAPI in zone B1 of the optic nerve. Note the morphology of CD68-positive activated microglia/macrophages (purple) compared with Iba1-positive microglia (green); they had a foamy appearance and were larger, rounded, and mainly located in the center of the cavernous zone, indicative of phagocytic activity, compared with the more elongated and ramified microglia (Figure 5B). Considering that Iba1 is a pan-microglial marker, it is possible that a portion of the CD68-positive cells are the same type as the Iba1-positive cells. In addition, Luxol fast blue (LFB) stain was used to evaluate axon myelination in longitudinal optic nerve sections. Sections from sham mice showed robust LFB staining with a relatively uniform distribution of nuclei (Figure 5C). In contrast, significant decreases in myelin intensity, as well as lesions containing increased numbers of cells, were observed in all injured mice (Figure 5C). Sections from high- and low-dose ST266-treated TBI mice appeared no different from sections from TBI-vehicle-treated counterpart animals.

We then examined the regions distant from primary damage (B2) with Iba1 and leukocyte common antigen 45 (CD45), two markers of microgliosis; amyloid precursor protein (APP), a marker of axonal injury; and glial fibrillary acid protein (GFAP), a marker of astrogliosis. Optic nerve sections from sham animals showed a baseline number of Iba1-positive microglia (Figures 5D–5G), whereas mTBI + vehicle mice exhibited extensive microgliosis (Figures 5I–5L). Sections from the mTBI mice treated with ST266 showed a significant decrease in microgliosis. No significant difference was observed between the low- and the high-dose-treated animals (Figure 5H). To further assess the presence of activated microglia and mature leukocytes, we chose CD45, a pan-leukocyte marker. Optic nerve sections from sham animals showed no CD45-positive microglia (Figures 5M–5P), whereas mTBI + vehicle mice exhibited an extensive cluster of activated microglia cells and T lymphocytes when their appearance showed single “donut-shape” CD45-positive nuclei (Figures 5R–5U). Comparable to effects on Iba1, both doses of ST266 treatment significantly reduced the burden of CD45-positive cells in the optic nerve (Figure 5Q). The three microglial makers (Iba1, CD68, and CD45) were chosen to evaluate microgliosis due to the diverse nature of the microglia population. Given that the microglia population in the brain is not uniform in shape, size, and function, several markers are needed to adequately characterize the microglial phenotype.

Healthy optic nerve sections have myelinated axons. The onset of demyelination in the optic nerve is associated with axon damage following blunt trauma such as mTBI. Optic nerve longitudinal sections from all groups were immunostained with APP, a marker of axonal injury. As expected, optic nerve sections from sham mice showed no sign of axonal injury (Figures 5V–5Y), whereas all injured animals revealed numbers of punctate, fragmented “axonal bulbs” (Figures 5AA–5DD). High-dose ST266 treatment showed a reduction in the number of axonal bulbs observed in the B2 region of the optic nerve sections (Figure 5Z).

### Treatment with ST266 reduced astrogliosis and microgliosis in the dorsal lateral geniculate nucleus (dLGN) and optic tract of animals with TON

**Figure 6:**
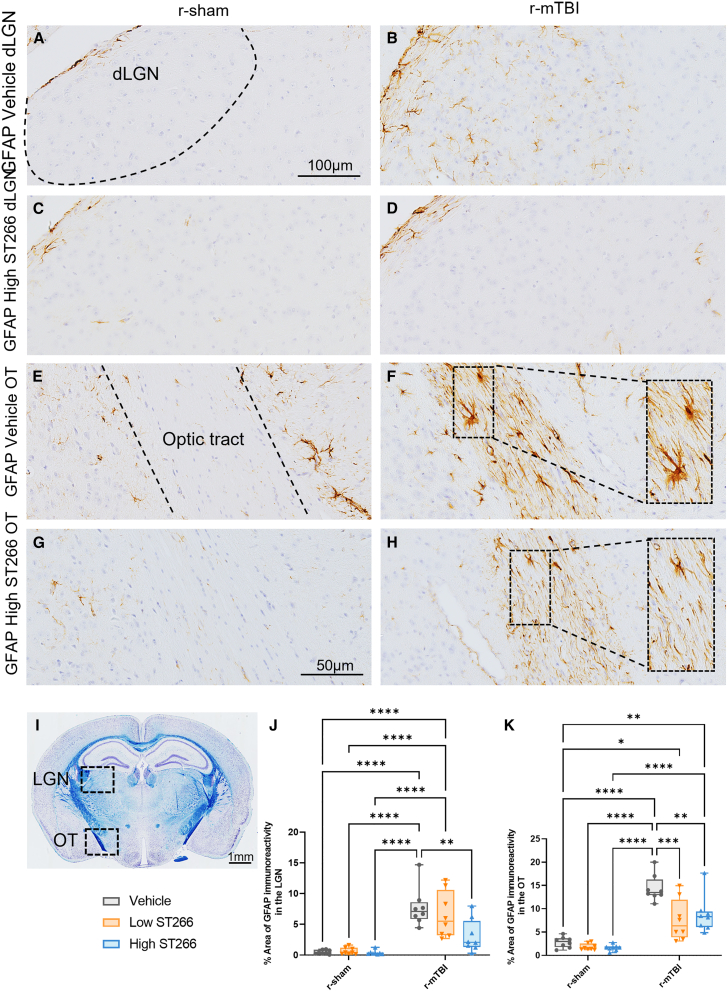
Treatment with a high dose of ST266 attenuated astrogliosis in the dLGN and optic tract Coronal sections containing (A–D) the dLGN and (E–H) the optic tracts were immunostained with GFAP to assess astrogliosis. The black dashed area highlights the location of the dLGN and the optic tract within the picture. (I) Luxol fast blue-stained mouse coronal sections containing the dLGN and optic tract. (J and K) GFAP immunoreactivity increased significantly in the injured group + vehicle compared with normal mice. Both low and high doses of ST266 reduced GFAP immunoreactivity in both the dLGN and the optic tract. A two-way ANOVA in the dLGN revealed an overall injury and treatment effect: injury F(1, 42) = 64.49, p < 0.0001; treatment F(2, 42) = 4.784, p = 0.0134. (F and H) In parallel, ST266 treatment reduced the size of fibrous astrocytes present in the optic tracts with an overall injury and treatment effect: injury F(1, 42) = 106.2, p < 0.0001; treatment F(2, 42) = 9.606, p = 0.0004. n = 8 mice per group. Tukey’s multiple comparisons are represented on the graphs ∗p < 0.05; ∗∗p < 0.001; ∗∗∗p < 0.0001; ∗∗∗∗p < 0.00001; box and whisker plots: min to max (all points are shown). Tissue sections were counterstained with hematoxylin.

**Figure 7:**
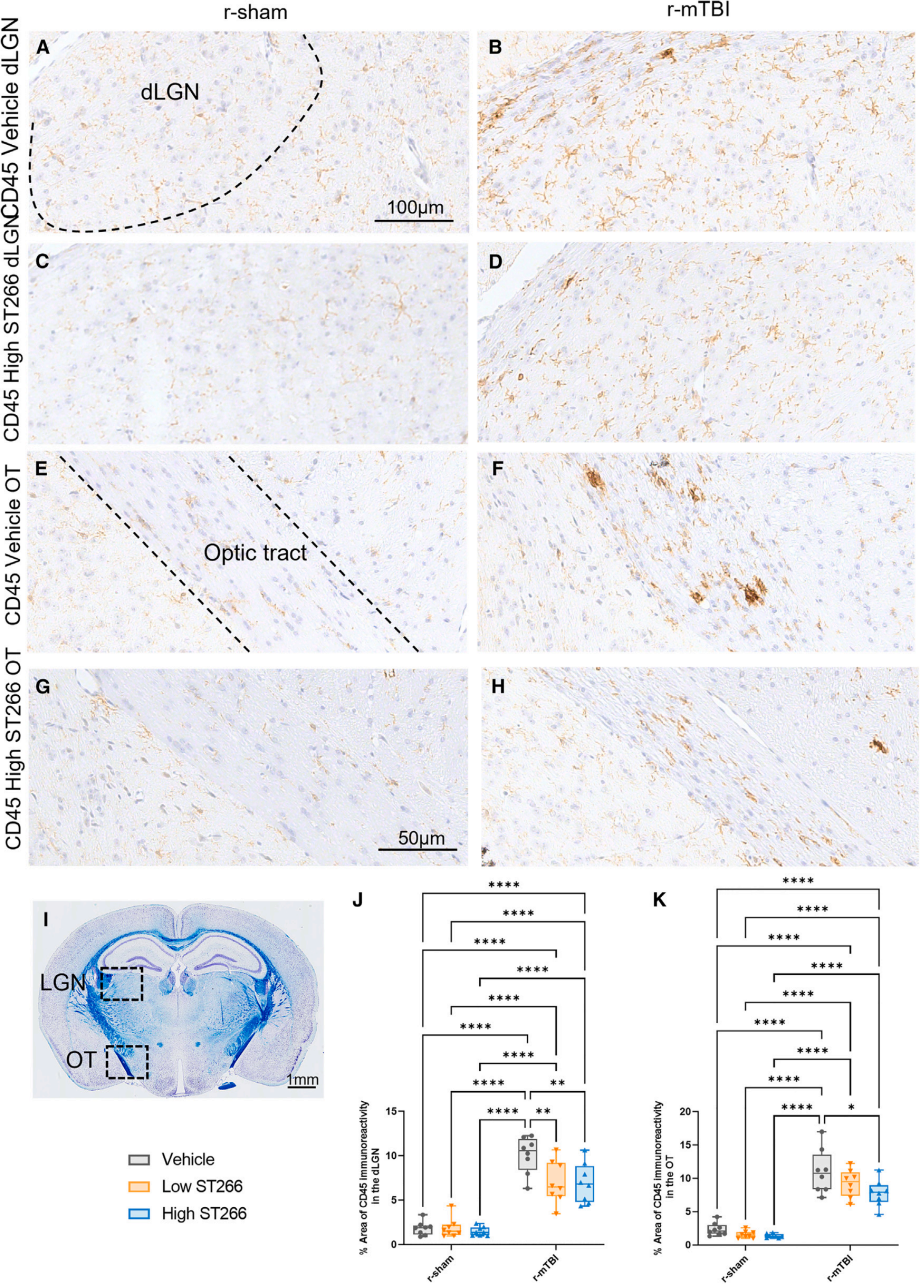
Treatment with a high dose of ST266 attenuated microgliosis in the dLGN and optic tract Coronal sections containing (A–D) the dLGN and (E–H) the optic tract were immunostained with cluster of differentiation receptors 45 (CD45) to assess microgliosis. The black dashed area highlights the location of the dLGN and the optic tract within the picture. (I) Luxol fast blue-stained mouse coronal sections containing the dLGN and optic tract. (J and K) CD45 immunoreactivity increased significantly in the injured group + vehicle compared with normal mice. Both low and high dose of ST266 reduced CD45 immunoreactivity in both the dLGN and the optic tract (F and H). A two-way ANOVA in the dLGN revealed an overall injury and treatment effect: injury F(1, 42) = 168.0, p < 0.0001; treatment F(2, 42) = 5.103, p = 0.0104. (F and H) In parallel, ST266 treatment reduced the size of fibrous microglias present in the optic tracts with an overall injury and treatment effect: injury F(1, 42) = 106.2, p < 0.0001; treatment F(2, 42) = 9.606, p = 0.0004. n = 8 mice per group. Tukey’s multiple comparisons are represented on the graphs ∗p < 0.05; ∗∗p < 0.001; ∗∗∗∗p < 0.00001. Box and whisker plots: min to max (all points are shown). Tissue sections were counterstained with hematoxylin.

The dLGN, a brain region that relays information from the retina to the visual cortex, and the optic tract, a continuation of the optic nerve, were evaluated for astrogliosis and microgliosis. Astrogliosis was measured by immunostaining with GFAP. Consistent with TBI pathology, GFAP immunoreactivity was increased among injured, vehicle-treated animals (Figures 6B and 6F). Treatment with either low-dose or high-dose ST266 reduced astrogliosis in the dLGN and optic tract of animals with TON (Figures 6D and 6H). Furthermore, treatment with ST266 reduced the size of fibrous astrocytes (Figures 6F and 6H). To evaluate microgliosis in the same brain regions, immunostaining with CD45 was performed. As expected, CD45 immunoreactivity was increased among injured vehicle-treated animals with TON (Figures 7B and 7F). Similar to the astrogliosis, microgliosis was reduced in the dLGN and optic tract of animals with TON treated with ST266 (Figures 7D and 7H). The size of fibrous microglia was also reduced (Figures 7F and 7H).

### Treatment with high-dose ST266 decreased the expression levels of the NLRP3 inflammasome

**Figure 8:**
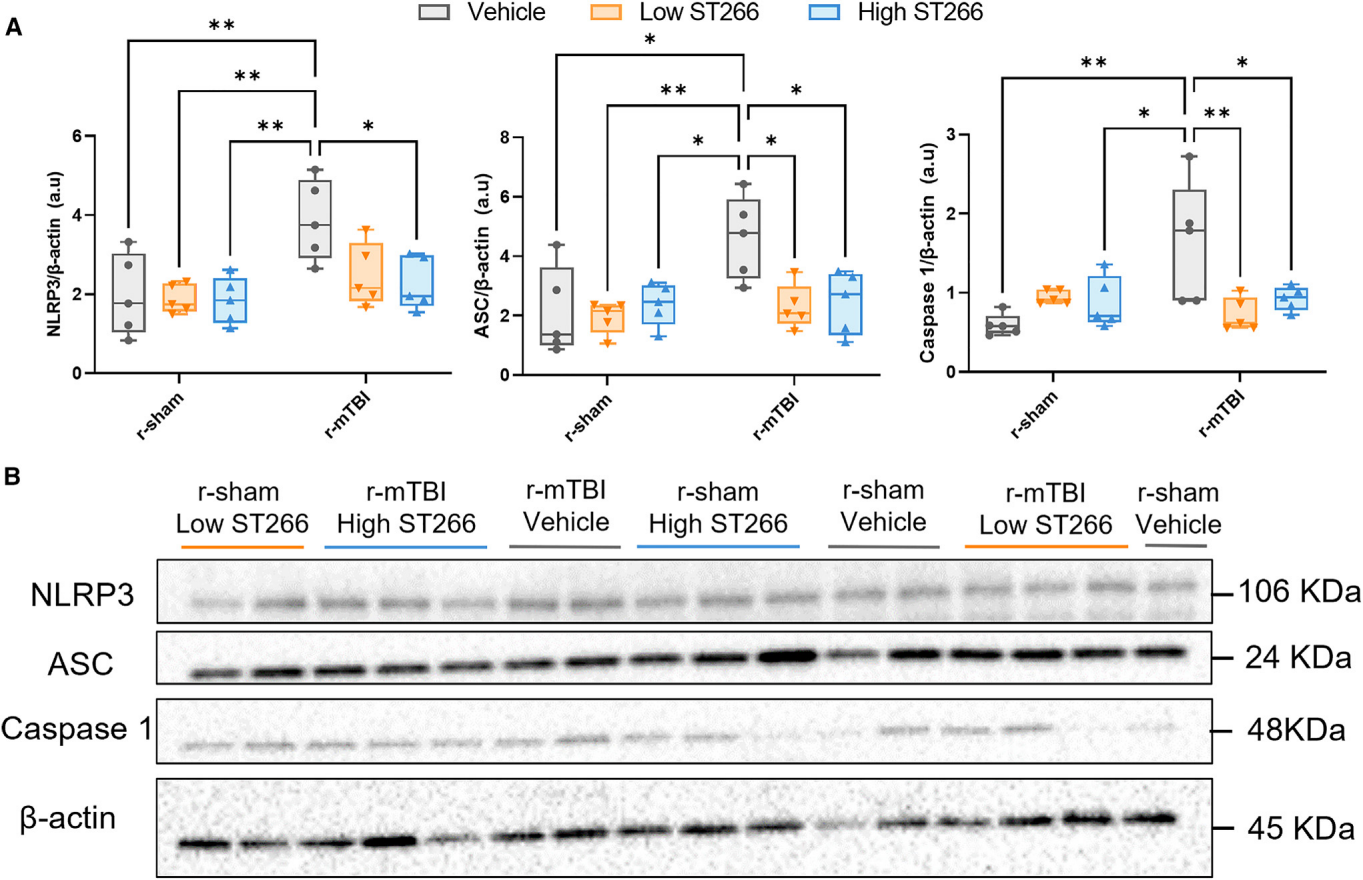
Western immunoblot analyses of inflammasome markers in cortical fractions at 18 days post-injury Representative images of Western blot bands shown in (B) were analyzed via densitometry to compile the graphs shown in (A). The expression levels of NLRP3, ASC, and caspase-1 were significantly increased at 18 days post-injury in the r-mTBI groups compared with the sham groups. A two-way ANOVA revealed an overall injury effect for ASC, injury F(2, 24) = 6.419, p = 0.0182, and caspase-1, injury F(1, 24) = 4.803, p = 0.0384, and a treatment and injury effect for NLRP3, injury F(1, 24) = 11.97, p = 0.0020; treatment F(2, 24) = 3.646, p = 0.0414. Values expressed as AU. All densitometry values for individual bands in (B) were normalized to the β-actin value for their respective lane and used for statistical analysis in (A); membranes were also normalized against one another. n = 5 mice per group. Tukey’s multiple comparisons are represented on the graphs ∗p < 0.05; ∗∗p < 0.001. Box and whisker plots: min to max (all points are shown).

The effect of ST266 treatment on the NLRP3 inflammasome was investigated. At 20 days post-injury, the expression levels of NLRP3, ASC, and caspase-1 were significantly increased among injured animals in comparison with the sham animals (Figure 8A). Treatment with high-dose ST266 led to a significant decrease in all markers among injured animals (Figure 8A). Treatment with low-dose ST266 led to a significant decrease in only ASC and caspase-1 (Figure 8A).

## Discussion

TON is a complication post-TBI resulting from inflammation in the optic canal, shearing injury, and biochemical inflammatory cascades.^32^ There are currently no means to prevent neuronal damage and vision loss in TON, and no existing methods can restore the function of the optic nerve or RGCs. A therapeutic option being explored preclinically is the use of neural progenitor cells (NPCs), which travel to the injury site and release an array of neuroprotective factors. Studies exploring the use of NPCs in rodent models of TON have found that NPCs promote the survival of RGCs and contribute to axon recovery.^33^^,^^34^ While NPC treatment has shown success in preclinical models, with all cell-based therapies there is a risk of rejection. To address this concern, cell-free therapies are being investigated. One promising cell-free therapy is the secretome from human stem cells, a secreted solution that contains cytokines and growth factors involved in wound healing, neuroprotection, anti-inflammation, apoptosis protection, and reduction of vascular permeability.^16^ ST266, the secretome from AMP cells, promotes an environment that fosters cellular protection and possible cell regeneration.^19^ In this study, we utilized intranasal delivery of ST266 in mice as a treatment for indirect TON sustained through repetitive closed-head injury. The protein components of ST266 are assumed to be delivered to the brain via the cribriform plate and olfactory nerves.^17^^,^^18^^,^^19^^,^^21^ We found that treating injured mice with ST266 improved spatial learning and memory, increased RGC density, and reduced markers of pathology in the optic nerve and the brain.

Examining spatial learning and memory through the Barnes maze revealed that animals with TON and treated with high-dose ST266 found the target hole quicker than untreated TON mice. Specifically, the performance of the r-mTBI/high ST266 group was similar to the level of performance displayed by the r-sham/high ST266 group. Furthermore, the number of exploratory pokes to the target hole was highest among the r-mTBI/high ST266 and r-sham/high ST266 animals. These data suggest that treatment with high-dose ST266 7restores spatial learning and memory in injured animals. The increase in performance among the r-mTBI/high ST266 group may be due to an increase in visual function, as the Barnes maze relies on the animals utilizing visual cues to successfully navigate the maze.^35^ However, when visual function was examined through optomotor testing, treatment with ST266 did not improve the optomotor response of injured animals (Figures S1 and S2).

RGCs are responsible for transmitting visual information from the eye to the brain. Notably, each RGC contains a long axon that contributes to forming the optic nerve and optic tract.^36^ These axons are vulnerable to damage and, when injured, can lose their ability to regenerate.^37^ TBI-induced TON is known to affect RGC viability, with low RGC density being observed in mice at both 3 and 10–13 weeks post-r-mTBI.^29^^,^^31^ These findings from our group have been confirmed by other laboratories using the same method^38^^,^^39^ or similar injury paradigms.^40^ Our current study reflected these findings, as RGC density was lowest among r-mTBI/vehicle animals at 20 days post-injury. However, among the injured animals who received a 10 day treatment of both high-dose and low-dose ST266, RGC density was significantly higher compared with the vehicle-treated animals. ST266 treatment has also been shown to preserve RGC density in other models, such as EAE.^18^^,^^19^ Considering that RGC loss is significantly greater at 13 weeks post-injury than 3 weeks post-injury,^30^ future investigation of the efficacy of ST266 treatment on chronic RGC loss is warranted.

Among the r-mTBI/vehicle group, damage throughout the ON was observed, with the most severe damage being present in area B1, the region of primary damage. In B1, increased cellularity was noted along with cavernous degeneration at 20 days post-injury. Our past studies have shown that the increased cellularity and cavernous degeneration persists as long as 10–13 weeks post-injury.^31^ Also present in area B1 of the r-mTBI/vehicle group was significant demyelination and reactive microgliosis noted by Iba1 immunoreactivity and the presence of CD68-positive foamy/phagocytic macrophages. Ten day treatment with ST266 did not ameliorate any of the pathological findings in area B1. In a different study investigating treatment for visual dysfunction post-TBI in a mouse model, treatment with raloxifene, a selective estrogen receptor modulator, did not alleviate microglial pathology in the area of primary optic nerve damage.^39^ These findings together indicate that damage to the primary damage site may be permanent and irreversible. However, our findings show that treatment with ST266 is effective in alleviating pathology in area B2, an area distal to the primary damage site. Specifically, both low- and high-dose ST266 treatment significantly reduced microgliosis in area B2 as indicated by a decrease in immunoreactivity of Iba1 and CD45. ST266 treatment also led to a decrease in axonal injury, demonstrated by a decrease in the number of axonal bulbs. Given the efficacy of ST266 treatment on the B2 region and not the B1 region, future studies should focus on developing treatment options for areas of secondary injury.

On a mechanistic level, secondary injury is due to the neuroinflammatory response and involves the activation of glial cells along with the release of pro-inflammatory cytokines. One component of this neuroinflammatory response is the NLRP3 inflammasome, and recent studies have investigated the NLRP3 inflammasome as a therapeutic target.^41^ We found that treatment with ST266 significantly decreased the expression of all three markers of the NLRP3 inflammasome, indicating the efficacy of ST266 in treating secondary injury.

To assess the effects of ST266 treatment further downstream of the primary damage site, astrogliosis and microgliosis were examined in the optic tract and dLGN. At 20 days post last injury, reactive astrogliosis was noted in both the optic tract and the dLGN of r-mTBI/vehicle animals. Astrogliosis in these brain regions has been shown to be present as early as 7 days post single closed-head injury^42^ and, in the optic tract, has been shown to persist for at least 6 months after repeated closed-head injuries.^43^ We also noted reactive microgliosis in the optic tract and dLGN. However, in a study examining microgliosis in the LGN 6 months post-injury, the LGN did not show signs of microgliosis.^43^ This may be due to the position of the LGN in the brain, as it is distal to the primary injury site. Treatment with ST266 attenuated astrogliosis and microgliosis in the optic tract and dLGN, as represented by a decrease in GFAP and CD45 immunoreactivity in these brain regions. Furthermore, ST266 treatment also reduced the size of both astroglia and microglia. These findings suggest that intranasal ST266 treatment is effective in treating not only optic nerve pathology, but also pathology in brain regions downstream of the primary injury site.

A limitation to this study is the acute time point of initiation of ST266 administration, as the clinical population may not receive treatment within 10 days of injury. Visual dysfunction resulting from mTBI may not develop until a chronic time point,^44^ and patients may wait for long periods of time before seeking treatment. An additional limitation is the use of only male mice, as mTBI outcomes differ among males and females.^45^^,^^46^^,^^47^ The effect of sex on visual dysfunction post-mTBI remains to be explored. Limitations regarding behavioral testing are also present. While the Barnes maze is a measurement of cognition, it relies on visual navigation. Considering that mice with visual impairments perform worse than controls on the Barnes maze task,^38^^,^^48^^,^^49^ an additional measure of cognition that is not solely reliant on the visual system would prove beneficial. To minimize the impact of visual impairments on Barnes maze performance, we utilized large spatial cue shapes whose smallest features correspond to an angular size of at least 15 min of arc as seen from the Barnes maze table, roughly equivalent to the size of each repeating pattern of black and white vertical stripes in the optomotor test at 0.25 CPD, the largest stripes presented to the mice in our assay. As a result, TBI-dependent optomotor impairments suggest that the smallest spatial cue features of the Barnes maze may be obscured by some loss of visual acuity, but this does not preclude the mice from visually locating the target hole by the use of larger spatial cue features present on the walls of the room. The lack of an ST266 effect on the optomotor results suggests that the improvements seen on the Barnes maze are not driven by differences in visual acuity between treated and untreated mice.

In this study, we have demonstrated the efficacy of intranasal ST266 in restoring spatial memory and learning and ameliorating pathological changes in the eye, optic nerve, optic tract, and dLGN in mice with TON. While ST266 treatment did not improve the extensive pathology present in the area of primary injury, it was effective in relieving secondary injury. In future studies, therapeutics that target secondary injury pathology are worth exploring, as treating the secondary injury may improve functional outcomes such as spatial memory and learning. In fact, clinical studies examining ST266 treatment in humans for corneal epithelial defects and gingivitis have shown that ST266 is well tolerated and effective.^23^^,^^24^ Given the efficacy of ST266 treatment for visual dysfunction demonstrated not only by our study but also by others,^17^^,^^18^^,^^19^ exploring the use of ST266 treatment in humans for TON is warranted.

## Materials and methods

### ST266

The novel secretome called ST266 was developed and produced under good manufacturing practice (GMP) conditions by Noveome Biotherapeutics (Pittsburgh, PA) using a proprietary population of cells known as AMP cells. The ST266 secretome product from these cells is proprietary as a result of the medium used to grow the cells, the culture conditions, and the specific of the AMP cell line used to produce the ST266. The AMP cells are cultured using a serum-free minimal medium in bioreactors and the conditioned culture medium is collected over several weeks. The culture medium is then further processed through filtration steps to remove certain cellular components and debris before storage of the bulk drug product. ST266 is then quality control tested to ensure lot-to-lot reproducibility using a series of tests that include a panel of quantitative ELISAs and purity and cell-based potency assays. Only lots that fall within the strict specifications of these rigorous tests are used for preclinical and clinical studies. The final drug product is devoid of extracellular vesicles due to confounding effects of the extracellular vesicles. The secretome extracellular vesicle removal methods are proprietary and are registered with the FDA in Noveome’s Drug Master File. The conditioned medium is ultrafiltered as part of the final manufacturing steps, with no cells or extracellular vesicles in the final product. ST266 contains analytes at nanogram and picogram concentrations similar to levels that naturally occur in the body.^20^

### Animals

C57BL/6J male mice were obtained from The Jackson Laboratory. They were housed in groups of three or four mice under standard laboratory conditions with a 12 h light/dark cycle. They had unlimited access to food and water for the duration of the study. All animal procedures were performed under institutional animal care and use committee approval and in accordance with the National Institutes of Health “Guide for the Care and Use of Laboratory Animals.”

### Injury groups and dosing schedule

A total of 72 mice were randomly selected for r-mTBI or sham injury with anesthesia exposure of the same duration and frequency (r-sham). Following our well-established model, injuries were administered every 48 h for a total of five injuries/sham exposures^25^^,^^26^^,^^28^ (Figure 2A). The rationale for this paradigm was to re-create the situation in which the brain undergoes multiple injuries within days or weeks of one another without adequate recovery time between injuries, as is often the case for contact sports and military operations. The dosing schedule consisted of either a low or a high daily dosing, the low dose being 5 μL in each nostril once daily and the high dose being 5 μL in each nostril *twice* a day, 10 h apart. Mice receiving vehicle received 5 μL of phosphate-buffered saline (PBS) in each nostril twice a day, 10 h apart. The mice received the treatment for 10 days starting at day 1 post last injury. The drug and vehicle were administered by injection into the opening of each nostril using a 10 μL pipette. A total of six groups were examined: low dose/r-mTBI, high dose/r-mTBI, vehicle/r-mTBI, and their r-sham counterparts. All 72 mice were then euthanized after completion of their last behavior test and the tissue was collected for biochemical and pathological analyses (Figure 2).

### Injury protocol

r-mTBI was administered using a mouse model of closed-head injury as previously extensively published by our group.^25^^,^^31^ Mice were anesthetized with 1.5 L/min of oxygen and 3% isoflurane for 3 min prior to the anesthesia or injury. Animals were placed onto a stereotaxic table with a heating pad set to 37°C, where the head was positioned between two rubber pads to limit lateral movement. A 5 mm blunt metal impactor tip was retracted and positioned midway relative to the sagittal suture before each impact. The injury was initiated using a MyNeuroLab controller with a strike velocity of 5 m/s and a depth of 1.0 mm and contact time of 200 ms. Once the mice righted themselves, they were removed from the stereotaxic table and placed into their home cages to recover further on a heating pad. Sham-injured animals underwent the same procedures and were exposed to anesthesia for the same length of time and frequency as the r-mTBI animals, but did not receive a hit, to control for the effects of the repeated anesthesia. To ensure consistency and repeatability of our animal model, injury administration was performed following the aforementioned protocol, thus ensuring mice were injured at the same location and with the same speed and depth of impact. Our several past studies have demonstrated consistent and repeatable results.^25^^,^^26^^,^^27^ Furthermore, the results obtained in our animal model have been confirmed independently by different labs.^38^^,^^39^

### Optomotor

Optomotor testing began with a 5 min acclimation period on a stationary plexiglass platform, 11.5 cm in diameter, elevated 19 cm above the center of the optomotor drum (29 cm in diameter), which was placed on a mechanically rotating table (Kopykake T1000 variable-speed turntable; Torrance, CA). The drum was covered with a set of stripes that filled the entire field of vision for each mouse. Stripe resolutions of 0.25, 0.52, 0.75, and 0.9 CPD were used, with one stripe resolution presented to each mouse during each trial. For each trial, the mouse was placed on the elevated stationary platform in the center of the drum for 5 min of acclimation. Following acclimation, the drum was rotated at 2 rpm in a clockwise direction for a period of 2 min. A video camera above the apparatus recorded the trial on video for manual assessment of the optomotor response by a blinded, trained observer. Following the completion of the first trial, the drum was stopped for a period of 30 s, followed by testing with the same stripes in a counter-clockwise rotation at 2 rpm for a further 2 min. Once completed, the mouse was removed and returned to its home cage, and the optomotor apparatus was cleaned using Quatricide (Pharmacal, Naugatuck, CT). Once each mouse completed testing, the stripe resolution was changed and the testing repeated for a total of eight trials per mouse (0.25, 0.52, 0.75, and 0.9 CPD stripe resolutions × 2 drum directions).

### Assessment of cognitive function

Spatial memory and learning were assessed using the Barnes maze behavioral test 24 h after the final treatment. Researchers conducting the experiments were blinded to groupings, and the Ethovision XT system was used to track the movement of each animal. Mice were given four trials per day for a period of 6 days, with each trial starting from one of four cardinal points in the maze. Mice were given 90 s to locate and enter the target box, and they were required to remain in the target box for 30 s prior to retrieval. On the 7th day, a single probe trial lasting 60 s was executed with a center starting point and the target box removed.

### Retina flat mount

All 72 animals were anesthetized with isoflurane and perfused transcardially with PBS (pH 7.4), followed by PBS containing 4% paraformaldehyde (PFA). After perfusion, the eyes were removed, injected with 4% paraformaldehyde, and then submerged in 1 mL of 4% PFA for 45–60 min. One eye from each mouse was dissected under a microscope to collect the retina. Once the sclera, lens, and vitreous body were removed, the retina was cut radially into four sections and resubmerged in 4% PFA for 40–50 min. After this time, the sections were permeated in 1 mL of PBS 0.5% Triton X-100 (PBT) for 30–50 min on a shaker. The retinas were then washed in 1 mL of PBS three times under agitation and then blocked in UltraCruz blocking reagent (sc-516214) for 1 h. After blocking, the retinas were no longer exposed to light. Brn-3a antibody (14A6) Alexa Fluor 488 was diluted in UltraCruz blocking reagent at a 1:200 ratio. The retina was then submerged in 500 μL of the prepared antibody overnight at 4°C. The next day the retinas were washed in PBS three times while covered in foil and agitated on a shaker before being mounted onto slides and cover glass with two drops of UltraCruz Hard-Set mounting medium with DAPI (sc-359850). The retinas were later imaged using a confocal microscope.

### Histology/immunohistochemistry

Animals were anesthetized with isoflurane and perfused transcardially with PBS (pH 7.4), followed by PBS containing 4% PFA. After perfusion, the brains were post-fixed in a solution of 4% PFA at 4°C for 48 h. The intact brains, optic nerves, and eyes were then blocked and processed as previously described.^25^^,^^31^ For each group, sets of optic nerve sections were cut. Sections were stained with H&E and LFB/cresyl violet (LFB/CV) using standard histological protocols. Sets of adjacent sections were stained for GFAP (1:20,000; Dako, Glostrup, Denmark; ZO334), Iba1 (1:5,000; Abcam, Cambridge, MA; ab5076), APP (1:20,000; Millipore, Billerica, MA; MAB348), CD45 (1:500; Cell Signaling, Danvers, MA; 70257), and CD68 (1:500; Abcam, Cambridge, MA; ab125212). As a negative control, for each antibody, a single section was processed for immunostaining without the inclusion of the primary antibody. Tissue sections were subjected to antigen retrieval with either heated tris-ethylenediaminetetraacetic acid (EDTA) buffer (pH 8.0) or citrate buffer (pH 6.0) under pressure for 7 min. Endogenous peroxidase activity was quenched with a 15 min H_2_O_2_ treatment (3% in water). Each section was rinsed and incubated with the appropriate blocking buffer (ABC Elite kit, MOM kit, Vector Laboratories, CA) for 20 min, before applying the appropriate primary antibody overnight at 4°C. The diluted biotinylated secondary antibody from the ABC Elite kit was then applied. Antibodies were detected using the avidin-peroxidase complex, after incubation with the chromogen 3,3-diaminobenzidine (DAB) peroxidase solution (0.05% DAB-0.015% H_2_O_2_ in 0.01 M PBS [pH 7.2]) for 6–7 min and counterstained with hematoxylin. IHC was performed with an antibody for APP (1:20,000). Prior to immunostaining, samples were deparaffinized in xylene and rehydrated through a gradient of ethanol solutions of decreasing concentrations (2× 100%, 95%, 70%). For antigen retrieval, slides were heated in a citrate 9 solution (pH 6.0) under pressure, washed with PBS, and transferred into a Sudan black solution (EMD Millipore, MA) (15 min) to inhibit autofluorescence. Before primary antibody treatment slides were blocked for 1 h with 10% donkey serum. The primary antibodies for Iba1 and CD68 were applied on the slides and left overnight at 4°C. The next day, donkey anti-goat IgG secondary antibody Alexa Fluor 488 was applied for Iba1 and goat anti-rabbit IgG secondary antibody Alexa Fluor 647 for CD68. Slides were mounted with ProLong Gold Antifade DAPI Mount. Fluorescence imaging was performed using a confocal microscope (LSM 800 Zeiss) at 20× and 63× magnification. Z stacks were recorded for every image and orthogonal projections were obtained to enable a 3D representation of the picture. Non-fluorescent samples were visualized with a bright-field microscope (BX60, Leica, Germany) and digital images were visualized and acquired using a MagnaFire SP camera (Olympus, Tokyo, Japan).

### Retinal ganglion cell count

For each whole mount (n = 6 per group), a series of nonoverlapping images were taken using a confocal microscope (LSM 800 Zeiss) at 20× magnification. Because of the difference in focal plane across the retinal preparation, focus stacking was performed to ensure that RGC nuclei were always in focus. The RGCs in the retina were analyzed by counting the number of Brn-3aY-positive cells within the central retinal area with ImageJ. Four images were acquired for each retina at 0.9 mm from the optic nerve head, one at each quadrant (inner images). After background adjustment, the images were converted to 8-bit gray-scale format and then adjusted for contrast, brightness, and threshold. Larger areas where the cells were perceived as fused were subdivided using the built-in watershed procedure. The RGC nuclei were counted with an automated counting algorithm in ImageJ^50^ within the cell nucleus size range of 10–300 μm^2^. To verify the quality of the analysis, automatically counted images were also evaluated individually for potential staining artifacts or debris on the slides that could interfere with the quantification.

### Immunohistochemical quantification in brain, eyes, and optic nerve

For each animal, brain (coronal sections, cut at 6 μm, n = 8), optic nerve (sagittal sections, cut at 6 μm, n = 8), and eyes (coronal sections, cut at 6 μm, n = 5) were stained and analyzed by an observer blinded to experimental conditions, using ImageJ software (US National Institutes of Health, Bethesda, MD, USA). Using this software, images were separated into individual color channels (hematoxylin counter-stain and DAB) using the color deconvolution algorithm. In this work, the optic nerve was analyzed topographically based on the consistent neuropathology resulting from our injury model,^29^ which has also been observed by many other research groups.^40^^,^^51^^,^^52^^,^^53^ The optic nerve was topographically segmented in three quadrants of 1,000 μm segments along the length of the nerve; the segments closest to the chiasm (B1) showed a significantly increased cellularity after injury compared with sham controls (Figures 5A–5C). Segment B2 was chosen, as it is anterior to B1 and ends at the approximate location of the optic nerve entry into the orbit canal.

Three non-overlapping areas of 100 μm^2^ for the optic tract (OT) were randomly selected, within which the area of GFAP/Iba1/CD45 immunoreactivity was calculated and expressed as a percentage of the field of view. Two non-overlapping areas of 150 μm^2^ in the visual cortex and one non-overlapping area of 100 μm^2^ in the dLGN were randomly selected, within which the area of GFAP/Iba1/CD45 immunoreactivity was calculated and expressed as a percentage of the field of view in coronal sections. The numbers of APP-positive profiles were counted and totaled in three non-overlapping areas of 200 μm^2^. Using ImageJ software, the average thickness of the corpus callosumwas calculated as previously described.^25^

### Biochemistry

The presence of NLRP3, ASC, and caspase-1 in homogenized cortical tissue was analyzed via western blot. The sensor, NLRP3; adaptor, ASC; and effector, caspase-1; are all components of the NLRP3 inflammasome.^54^ The NLRP3 inflammasome is a critical component of the innate immune system in response to cellular damage caused by TBI.^55^ Given the established link between the NLRP3 inflammasome and TBI, NLRP3 holds potential as a therapeutic target.^41^ As such, we investigated the effect of ST266 treatment on the NLRP3 inflammasome. Western blot analysis was performed on 30 cortical samples with five mice representing each group. The cortex was submerged in lysis buffer and homogenized using a sonicator for 10 s, three times, resting on ice in between each two sonications. Equivalent total protein amounts were analyzed by sodium dodecyl sulfate-polyacrylamide gel electrophoresis under denatured and reduced conditions using 4%–20% TGX stain-free precast gels (Bio-Rad) and electroblotted onto a low-fluorescence polyvinylidene difluoride (PVDF) membrane for 2 h at 90 mA constant current. Membranes were then washed in deionized water and blocked for 1 h at room temperature with 5% non-fat milk in Tris-buffered saline (TBS). Membranes were then incubated with primary antibodies overnight at 4°C. The following primary antibodies were used at the given concentrations: caspase-1, 1:100 (Cell Signaling, Danvers, MA; 24232S); ASC/TMS1, 1:1,000 (Cell Signaling, Danvers, MA; 67824S); NLRP3/NALP3, 1:1,000 (Adipogen, San Diego, CA; AG-20B-0014-C100); and β-actin, 1:1,000 (Santa Cruz, Dallas, TX; SC-47778). Membranes were washed with deionized water, incubated with the appropriate secondary antibody for 1 h at 4°C, washed once more, and developed using ECL chemiluminescent detection reagent (GE Life Sciences). Membranes were imaged using a Bio-Rad ChemiDoc western blot imager, and densitometry results of individual bands were collected using ImageLab 5.2 (Bio-Rad) software. Target protein values for each lane were normalized between each two membranes and against densitometry values for the respective β-actin value.

### Statistical analysis

All behavioral and pathological data were analyzed using GraphPad Prism 9.0 (San Diego, CA, USA). Data were tested for normality using the Shapiro-Wilk W test; when not normally distributed, the data were transformed using square-root or natural log transformation. If the data were still not normal after transformation, non-parametric methods were used for analysis. Behavioral experiments were analyzed using two-way ANOVA, repeated-measures ANOVA, and PERMANOVA for search strategy quantification. Repeated-measures ANOVA was used to compare performance during the 6 days of acquisition of the Barnes maze between the matching injury groups when the data were normally distributed. Potential sphericity violations were corrected by adjusting degrees of freedom for all repeated-measures effects by using the Greenhouse-Geisser estimate for epsilon. Probe, elevated plus maze, and quantitative histologic parameters were analyzed with two-way ANOVA, with a Tukey’s *post hoc* correction for multiple comparisons, unless indicated. Only p values <0.05 were statistically significant and are indicated by asterisks in the figures.

### Data availability

The datasets from the current study can be made available upon reasonable request.

## Supplemental information

Document S1. Figures S1 and S2

Document S2. Article plus supplemental information
