# Supplementary material for: Human amnionic progenitor cell secretome mitigates the consequence of traumatic optic neuropathy in a mouse model
Source: Mol Ther Methods Clin Dev. 2023 Apr 18;29:303–18. doi: 10.1016/j.omtm.2023.04.002 (PMC10285248; doi:10.1016/j.omtm.2023.04.002)
Supplement: Document S1. Figures S1 and S2 [file mmc1.pdf]

OMTM, Volume *29*

Supplemental information

Human amnionic progenitor cell secretome mitigates the consequence of traumatic optic neuropathy in a mouse model

Robyn McCartan, Arissa Gratkowski, Mackenzie Browning, Coral Hahn-Townsend, Scott Ferguson, Alexander Morin, Corbin Bachmeier, Andrew Pearson, Larry Brown, Michael Mullan, Fiona Crawford, Radouil Tzekov, and Benoit Mouzon

Optomotor Reflex Response Frequency

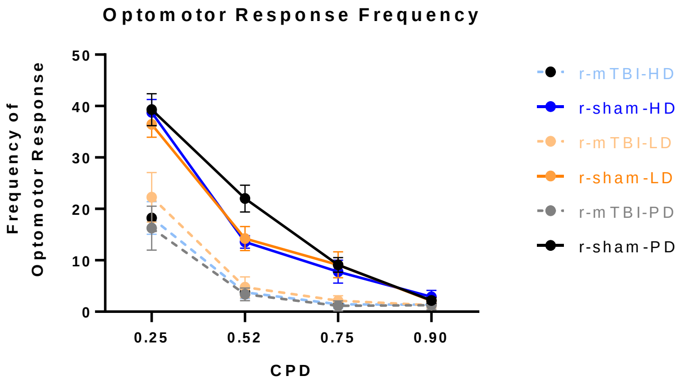

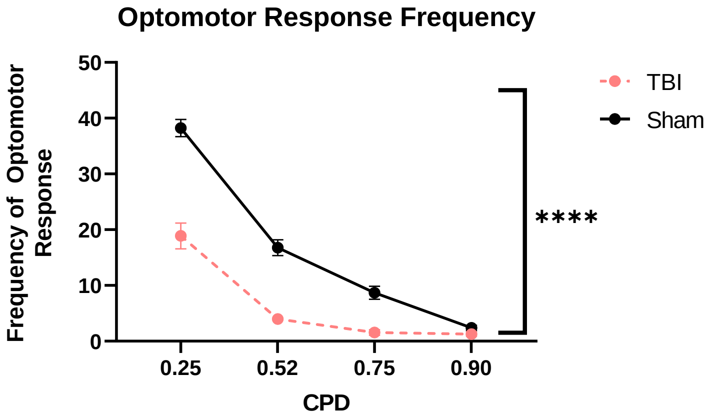

Two-way RM ANOVA

Source of Variation	% of total variation	P value	P value summary	Significant?
CPD x Injury	6.089	<0.0001	****	Yes
CPD	56.90	<0.0001	****	Yes
Injury	13.62	<0.0001	****	Yes
Subject	6.775	0.0584	ns	No

Figure S1. Optomotor response was assessed using repeating stripes at a speed of 2 revolutions per minute (rpm). Stripe thickness varied from 0.25 to 0.9 cycles per degree (CPD). The optomotor response was assessed and counted by a trained and blinded observer. Sham and injury groups were pooled for statistical analysis of the initial cohorts. The optomotor response frequency significantly decreased with increasing CPD and in response to injury ($p<0.0001$).

Duration of the Optomotor Response

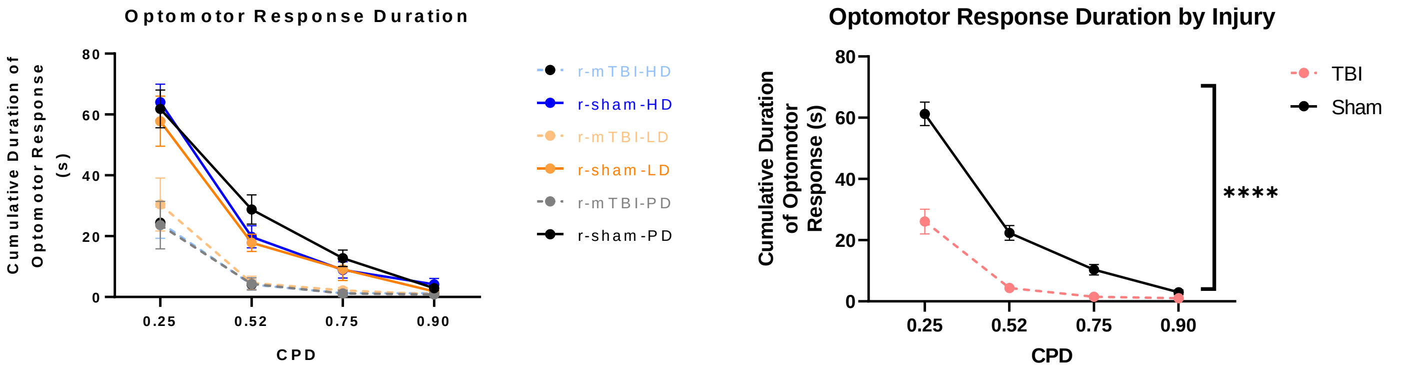

Two-way RM ANOVA

Source of Variation	% of total variation	P value	P value summary	Significant?	Geisser-Greenhouse's epsilon
CPD x Injury	7.478	<0.0001	****	Yes	
CPD	51.70	<0.0001	****	Yes	0.4583
Injury	12.34	<0.0001	****	Yes	
Subject	8.362	0.0563	ns	No	

Figure S2. Optomotor response was assessed using repeating stripes at a speed of 2 revolutions per minute (rpm). Stripe thickness varied from 0.25 to 0.9 cycles per degree (CPD). The optomotor response duration was quantified by a trained and blinded observer. Sham and injury groups were pooled for statistical analysis of the initial cohorts. The optomotor response duration significantly decreased with increasing CPD and in response to injury ($p<0.0001$).
